# Exploring the process of making health behaviour changes in traditional acupuncture: a longitudinal qualitative study

**DOI:** 10.1080/21642850.2026.2709724

**Published:** 2026-07-28

**Authors:** Jonquil Pinto, Katherine Bradbury, Dave Newell, Felicity Bishop

**Affiliations:** a School of Psychology, Faculty of Environmental & Life Sciences, University of Southampton, Highfield Campus, Southampton, United Kingdom; b Faculty of Medicine, University of Southampton, Highfield Campus, Southampton, United Kingdom; c Health Sciences University, Parkwood Campus, Parkwood Road, Bournemouth, United Kingdom

**Keywords:** Acupuncture, complementary therapies, health behaviour, health promotion, qualitative research

## Abstract

**Background:**

Health behaviours (e.g. exercise/diet/alcohol/smoking) are a major public health concern and traditional complementary and integrative medicine providers such as traditional acupuncturists could make an important contribution. We explored the support for health behaviour change provided to and experienced by traditional acupuncture patients to understand the ways in which traditional acupuncturists may contribute to their patients making changes to their health behaviours.

**Methods:**

Longitudinal qualitative research methods were used to capture experiences as they change over time with nine patient‒practitioner dyads (8 female patients and 1 male, aged 23–55, and 3 female traditional acupuncturists, aged 35–63). Consultations were audio-recorded and in-depth qualitative interviews were conducted with patients (twice) and traditional acupuncturists (once). Experiences were explored with primary focus on the patients; data were coded both deductively and inductively and themes were produced using reflexive thematic analysis.

**Results:**

Patients at different stages of readiness to change need different types of support—those overwhelmed by symptoms/stress needed help to gain control, patients more ready to change needed help sustaining motivation. Acceptance of lifestyle/behaviour advice was related to establishing a cycle of trust, in practitioner, explanations and treatment. Decisions to enact behaviour change were based partly on changes in feelings/mood as well as reasoned understanding (e.g. of behavioural contribution to symptoms).

**Conclusions:**

Patients' response to behaviour change support in traditional acupuncture may vary according to their symptom control and stress. For some, support for symptoms/stress may need addressing before any behaviour change intervention. Therapeutic trust, coherent explanations and positive feeling from treatments were important in supporting change. These findings link to concepts from the trans-theoretical model and stages of change, social cognitive theory, and the common sense model of self-regulation and dual process theories of behaviour.

## Introduction

Promotion of healthy behaviours has become a key public health aim (Public Health England, 2016, 2019). Traditional, Complementary and Integrative Medicine (TCIM) providers' role in the promotion of health behaviour change has been identified as potentially important (Hawk et al., 2012; Royal Society for Public Health & Professional Standards Authority, 2017), but psychological behaviour change research has not fully examined the experiences or outcomes of TCIM patients. TCIM users have a high prevalence of chronic disease and modifiable behaviours; prevention of disease and health maintenance are key reasons for attending TCIM; and TCIM typically involves high levels of patient‒personnel contact with repeated visits of long duration (Bishop et al., 2019; Davis et al., 2011; Williams-Piehota et al., 2011).

### Traditional acupuncture

Traditional acupuncture is one TCIM therapy shown to address patients' lifestyle and health behaviours (Pinto et al., 2021). Traditional acupuncture users report seeking treatment for disease prevention (Austin et al., 2015) and use is associated with healthy behaviours: individuals engaged in regular physical activity, ex-smokers and non-smokers are more likely to be traditional acupuncture users (Upchurch & Rainisch, 2014).

Traditional acupuncture is acupuncture practiced using a framework based on traditional Chinese/East Asian medicine. While there are several distinct frameworks which differ from each other in specific details, they tend to share the common characteristic of approaching health and illness from a holistic viewpoint based on traditional, pre-scientific models. These approaches tend to connect mind-body-lifestyle-environmental factors to inform diagnosis, treatment and advice. This is distinct from Western medical acupuncture, which is understood primarily in terms of the physiological mechanisms and outcomes of needling (White, 2009).

Traditional acupuncture is regarded as a complex intervention (Petticrew, 2011; Skivington et al., 2021; 2024) containing multiple components that interact with each other including acu-point needling/stimulation, contextual elements (e.g. therapeutic relationship), individualised treatments and lifestyle change advice (Bishop et al., 2021; MacPherson et al., 2006).

A survey of 352 British Acupuncture Council (BAcC) members showed that 57.7% of patient visits included some health behaviour change support (Pinto et al., 2022). A Critical Interpretive Synthesis review of 40 studies found qualitative evidence that patients make health behaviour changes during treatment programmes (e.g. Evans et al., 2011) and suggested possible mechanisms of change within traditional acupuncture which support behaviour change. Specific elements of treatment which may promote behaviour change included: individualised advice (i.e. linking behaviour change advice to diagnosis and to the specific symptoms which concern patients); holistic/biopsychosocial explanations (i.e. providing an explanatory model which explicitly connects behaviour, psychological elements and disease processes); therapeutic relationship (i.e. establishing a strong therapeutic partnership); simultaneous treatment of behaviour-limiting symptoms (i.e. through acu-point stimulation/needling to treat symptoms such as pain or nicotine withdrawal which may be a barrier to healthy behaviours); and patients' physical involvement with intervention (i.e. experiencing a somatic treatment while also being given verbal support) (Pinto et al., 2021). However, this synthesis was limited due the small number of qualitative studies which explicitly focussed on how behaviour change occurs in traditional acupuncture and highlighted the need for more in-depth analysis.

### Approaches to health behaviour change in mainstream healthcare

Brief interventions to support healthy behaviours are widely adopted across mainstream healthcare; however, evidence for effectiveness is sparse, partly due to a lack of high-quality research assessing impact of these interventions on health behaviours (Parchment et al., 2021). Available evidence suggests impact of brief interventions may be limited, with mainly short-term effects on behaviours and very short interventions (less than 5 minutes) may be less effective than longer interventions or more intense interventions over longer periods (SCHARR Public Health Collaborating Centre, 2012).

Digital health interventions (DHIs) for self-management of specific conditions have an important role in improving health behaviours (Allegrante et al., 2019). However, there is a problem in low uptake and engagement with DHIs among some patient groups (Kohl et al., 2013). For some patients, engagement with behaviour change interventions is connected to receiving face–face support and having a sense of relatedness with health care providers (Lie et al., 2017).

Similarly, evaluations of social prescribing programmes have found patients/clients report too short consultation time and a need to develop trusting relationships with those providing support, as well as needing support which addresses understanding and attitudes towards health/activity and physical capability (Polley & Sabey, 2022).

Findings from brief interventions, DHIs, and social prescribing studies suggest it may be worth exploring alternative approaches to individual-level behaviour change interventions, particularly those which emphasise strong therapeutic relationships, with longer consultation times, and over longer periods. The existing studies of traditional acupuncture suggest that the approach of these practitioners may offer a method of supporting behaviour change and healthy lifestyles which emphasises some of these elements. The distinct approach may address some of the shortcomings in existing mainstream interventions. However, we need to understand more clearly how behaviour change support is provided in traditional acupuncture and how patients experience and respond to this support. Understanding these experiences can help to elucidate the processes of health behaviour change, which is important because mechanisms remain somewhat unclear and are often overlooked in trials (Hagger et al., 2020).

## Study aims/objectives

The current study aimed to explore how health behaviour change support is offered by traditional acupuncturists and how it is experienced by patients.

The objectives were to: explore the process of change; identify elements which traditional acupuncture patients experience as enabling behaviour change; and understand how particular clinical approaches/techniques and psychological processes lead to behaviour change.

## Methods

### Qualitative approach and research paradigm

To develop understanding and build theory, the study design aimed to create a direct window onto the acupuncturist–patient interaction and the subjective reflections of participants. Longitudinal qualitative research provides a method of capturing experiences as they change over time, exploring behavioural transitions in a way which is difficult in cross-sectional studies (Tuthill et al., 2020). We used multiple perspectives from patient‒acupuncturist dyads with a focus on the experience of patients, collecting data in the form of recordings of every consultation/treatment for each patient over consecutive weeks as well as two in-depth interviews with each patient and one in-depth interview with each traditional acupuncturist. Online video interviews were semi-structured, consisting of open-ended questions to obtain rich data and allow the researcher and participant to co-construct understanding and knowledge.

This research is undertaken from a pragmatist position which places emphasis on the implications of the outcomes of research methods and questions. The aim of the research is to provide answers which have valuable consequences for real-world problems (Legg & Hookway, 2021). The pragmatic approach emphasises the importance of keeping open various types of inquiry (Legg & Hookway, 2021). Taking a pragmatic approach allows the use of whichever epistemological tools are best able to provide meaningful, useful answers for a given question.

### Context, setting and participants

The objectives defined the context as the practice of traditional acupuncturists. We selected to sample participants from the BAcC as this is the main professional body of traditional acupuncturists in the UK and many members had taken part in a previous survey study and had previously consented to be contacted for research purposes.

#### Recruitment

Acupuncturists were purposively sampled with the aim of reaching depth of understanding, selecting those who indicated in a previous study that they are engaged in behaviour change work. We looked at a combination of their survey responses in a previous study (Pinto et al., 2022) to identify a variety of acupuncturist participants who offer support for behaviour change ‘always’, ‘most of the time’ and ‘sometimes’. We compiled a list of 38 who said ‘always’, 39 who said ‘most of the time’ and 34 who said ‘sometimes’. We initially contacted 15 from the ‘always’ list, 15 from the ‘most of the time’ list and 15 from the ‘sometimes’ list. Six replied to our email request, 2 declined to take part after receiving further information due time constraints and personal reasons and 1 did not reply. Three consented to take part. These practitioners were asked to recruit patient participants by inviting consecutive new patients attending their clinic for treatment until 3 patients were recruited per practitioner. Recruitment records for patients were limited as we relied on practitioners to conduct this recruitment.

#### Traditional acupuncturists

BAcC members registered as ‘practicing members’ were eligible. Exclusion criteria: Those unlikely to be able to complete the study (including participation in interviews) or unable to speak English language (due to resources).

##### Patients.

Each traditional acupuncturist recruited at least three patients starting a new course of treatment. Exclusion criteria: We excluded potential participants if they were under 18, unable to speak English language (due to resources), unlikely to be able to complete the study (including participation in interviews), and people with alcohol/substance abuse dependence disorders. We made the decision to exclude these patients in order to specifically to focus on health behaviour change rather than addiction, however this limits the transferability of the findings to these vulnerable groups.

Participants were offered a £ 20 John Lewis gift voucher incentive.

#### Setting

The traditional acupuncturists and patients were in private (paid for) treatment settings which is typical of this therapy in the UK. This was a pragmatic study in which individualised, holistic care was provided by the traditional acupuncturists as per their usual practice.

#### Researcher/participant relationship

There was no prior relationship between the researchers and participants.

#### Sample size

The sample size was guided by the concept of ‘information power’. This approach considers the amount of information within a sample that is needed for answering the specific research questions (Malterud et al., 2016). Information power was used given the narrow study aim, dense longitudinal data, dyadic design, repeated interviews, consultation recordings, strong dialogue quality and theory-informed analysis. The participants in this study were a specific group and some theory about behaviour change in this context existed (Pinto et al. 2021). Analysis was conducted concurrently with data collection and when a rich set of codes and themes was generated to address the research questions, the research team agreed that sufficient data had been collected. In application, the research team repeatedly discussed the analysis and themes in relation to the aims of the study. We decided to stop collecting data when sufficient data and analyses had provided a deep interpretation of the data to meet our aims.

### Consent

Participant information forms were given to those interested in taking part in the study (see Additional Files 1 and 2). A signed consent form was emailed to the researcher by all participants (see Additional Files 3 and 4) and consent was confirmed verbally at the beginning of each interview and consultation.

### Reporting standards

Reporting adhered to the Standards for Reporting Qualitative Research (SRQR) (O’Brien et al., 2014).

### Data collection and processing

#### Interviews

Patient interviews were conducted after two weeks of treatment to explore their experience at the beginning of the treatment programme when lifestyle/behaviour may have just begun to be discussed. This time-point was decided based on survey findings that reported traditional acupuncturists frequently start to discuss lifestyle change in the second session of treatment (Pinto et al., 2022) A second interview was conducted at least six weeks after the first consultation to explore the patient's experiences of any changes over time, after the therapeutic relationship may have developed and a short programme of traditional acupuncture treatment had been received. Using repeated interviews over time alongside consultation recordings generates a large volume of data suitable for a focus on a small number of cases in considerable detail (Malterud et al., 2016). Traditional acupuncturist interviews were completed after their three patients had finished their treatment programme or after six treatments. Interviews were online with cameras on when possible and recorded using Microsoft Teams (Microsoft Corporation, 2016). Interviews were semi-structured and lasted between 15 and 40 minutes. The interview guides were informed by prior literature (Pinto et al., 2021; 2022) and then refined by a multidisciplinary research team including an acupuncturist, two health psychologists and one CM researcher (no participants were involved in designing the interview guide). Semi-structured topic guides explored experiences of treatment, perceptions of lifestyle/behaviour discussions, barriers and facilitators to change, therapeutic relationships, emotional and symptom changes, maintenance of behaviour changes over time, and perceptions of helpful or unhelpful aspects of treatment. (See Additional Files 5 and 6).

#### Consultation recordings

Traditional acupuncturists were sent digital recording equipment and instructions. Consultations were then recorded with patient participants from their initial consultation and up to eight following appointments. Digital files were sent to the research team using the host institution's secure encrypted file transfer service. Data were collected between February and August 2022.

#### Missing data

Two consultation recordings failed, one interview was missed and three interview recordings only provided audio data.

#### Transcripts and data coding

All recordings were viewed/listened to in full. We used theoretically purposive transcription in which excerpts were selected systematically using selection criteria tied to research aims. All sections of consultations relating to lifestyle habits, changing behaviour, health behaviour, and experiences of support for behaviour change were transcribed. The main sections of talk we did not transcribe were those related to scheduling appointments, talk in relation to the needling part of treatment, and incidental chat (e.g. weather/holidays). No relevant sections were intentionally excluded. In practice, this was an iterative analytic selection process, moving back and forth between early analysis and new selection. All interview recordings were transcribed.

Transcripts auto-generated from Teams were initially corrected and coded while listening to and watching the video recordings, so salient sections were able to be considered more completely and nuances of speech helped clarify the text. Initial coding in NVivo (QSR International Pty Ltd., 2020). enabled each code to be linked to single or multiple data sources. As more data was added codes were refined and extended.

After the coding process, the list of codes and related excerpts was copied to Excel (Microsoft Corporation, 2018) to enable a flexible re-organisation of codes into themes as analytical thinking progressed. As themes were further developed, the key concepts were mapped out with pen and paper, using thematic mapping as an analytic technique. The development of themes was completed by working back and forth between Excel and paper and re-listening to audio/video to check themes remained grounded in original data.

Data were anonymised through use of pseudonyms and data were stored in secure folders within the University system.

### Data analysis

As the aim was to gain deep insights and to identify patterns of lifestyle change in traditional acupuncture across the dataset, the analysis focussed on achieving an in-depth understanding within and across cases. Coding of units of meaning was completed both inductively and deductively, drawing on concepts from existing theories of behaviour and behaviour change (see Table 1).

Reflexive thematic analysis (Braun & Clarke, 2006, 2019) was used to explore experiences of both traditional acupuncturists and patients, with primary focus on the patient; therefore, the consultations and interviews relating to each patient were analysed together. This enabled an exploration of the processes involved in supporting and making health behaviour change for each individual, to gain a deep insight into how patients experienced behaviour change support, how change occurred (or why it did not) and how this corresponded with traditional acupuncturists' strategies, approach and techniques.

Initial inductive coding grounded in participant accounts was followed by interpretive engagement with behaviour-change theories. We used theories as interpretive lenses, not fixed coding frames: Phase 1 Inductive coding grounded in participant accounts; Phase 2 Cross-case/theme development; Phase 3 Interpretive engagement with behaviour change theories.

JP completed the interviews and led the coding and then discussed the data/themes in depth with the research team. Themes were finalised through discussion and were further interpreted with reference to existing behaviour change theories (see Discussion) to make conceptual links to the key themes produced in our findings.

The findings were returned to participants for comments before publication.

### Researchers, reflexivity and trustworthiness of data

FB and KB are health psychologists and DN is TCIM researcher. JP trained in qualitative methods during her PhD, FB has over 15 years' experience of leading qualitative studies and KB over 10 years. JP has trained and worked as a traditional acupuncturist. Attempts to support reflexive awareness of researcher assumptions were made, including producing interview guides (Additional Files 5 and 6) in advance with the whole research team, to ensure questions were framed to reveal both negative and positive responses and that prompts were included to fully explore issues raised by each participant. Also, the researchers' own views on this topic were explored through use of memos and discussion with the research team following interviews. In developing themes we used participants' language to ensure that meanings were rooted in data.

**Table 1.** Thematic map – showing development of inductive and deductive codes into themes.

Theme	Code	Inductive/deductive (source)
From gaining control to keeping up motivation: different stages need different support	Fear and confusion re illness	Inductive
	Symptoms overwhelming (cognitive and emotional burden)	Inductive
	Physical and emotional/psychological obstacles (lack of psychological capability)	Deductive see The Behaviour Change Wheel on psychological capability (Michie et al., 2011)
	Experience of stress reduction/emotional regulation within traditional acupuncture treatment	Inductive
	Negative emotional associations with health behaviours	Inductive
	Social/environmental obstacles	Inductive
	Self-efficacy improvement	Deductive see Social Cognitive Theory (Bandura, 1991).
	Expectation of lifestyle change support	Inductive
	Negative response to advice/resistance	Inductive
	Negative coping behaviours	Inductive
	Priority on improving negative affective state before behaviour	Inductive
	Encouragement/Practitioner efficacy aids motivation	Inductive
Trust and acceptance – includes three subthemes: trust in therapist, trust in explanations, trust in treatment	Rapport	Inductive
Therapist	Communication style—non-judgemental/respect for autonomy	Inductive
	Exploring patients ways of being/drivers/motivations	Inductive
	Being known	Inductive
	Intrusion/Permission	Inductive
	Long consultations	Inductive
	Patient perception/relationship with practitioner linked to how much they accept explanations	Inductive
Explanations	Explanatory framework/holistic model with broad application	Deductive see Logic Model of Behaviour Change in Traditional Acupuncture (Pinto et al., 2021)
	Illness identity/coherence	Deductive see Extended Common Sense Mode (Hagger & Orbell, 2022)
	Buying into holistic health framework (supported by therapy/therapist buy in)	Inductive
	Using explanatory framework (TCM/5 elements) to promote self-regulation (re-framing, mind-set)	Inductive
	Links to how behaviour can improve their symptoms	Inductive
Treatment	Feels well from treatment	Deductive see Logic Model of Behaviour Change in Traditional Acupuncture (Pinto et al., 2021)
	Positive effect of treatment on stress	Deductive see Logic Model of Behaviour Change in Traditional Acupuncture (Pinto et al., 2021)
Changes in feelings: mood, calm, positivity and ‘just feeling like it’.	Physical involvement—awareness of body states	Deductive see Logic Model of Behaviour Change in Traditional Acupuncture (Pinto et al., 2021)
	Choices based on immediate feelings	Inductive
	Feeling more positive in mood makes lifestyle change easier	Inductive

### Ethics statement

Ethical approval was obtained from the University of Southampton, School of Psychology Ethics Committee (reference 68350).

## Findings

### Participant characteristics

The three traditional acupuncturists were female, aged between 35 and 63 years, in practice for between 5 and 19 years, practicing a traditional form of acupuncture (such as traditional Chinese medicine) and working in the private sector. The nine patient participants included 8 females and 1 male, between the ages of 23 and 55. All participants were based in the United Kingdom (England and Scotland). Reasons for seeking treatments included pain, fertility, sinus congestion and anxiety. Patients attended between two and eight treatment sessions during the study, typical for a course of traditional acupuncture (Vickers & Zollman, 1999). Table 2 presents details of participants' characteristics and participation.

**Table 2.** Patient participants' characteristics.

Pseudonym	Age range	Gender	Presenting condition	Acupuncture sessions attended	Interview completion
Amelie	18–24	F	Anxiety	6	2
Cara	35–44	F	Fertility	6	2
Stephanie	25–34	F	Fertility	4	2
Adam	35–44	M	Back pain	3	2
Isobel	25–34	F	Neck and back pain	2	2
Sam	45–54	F	Muscular pain	8 (2 recordings missing)	2
Priti	25–34	F	Bowel disease	6	2
Gillian	55–64	F	Sinusitis	6	2
Kelly	35–44	F	Fertility	4	1

### Main themes

Reflexive thematic analysis generated three major themes from the data that explain the process of behaviour change within these clinical encounters: *From gaining control to keeping up motivation – different stages need different support*; *Trust in a) therapist, b) explanations and c) treatment*; *Changes in feelings – mood, calm, positivity and ‘just feeling like it’*.

### From gaining control to keeping up motivation: different stages need different support

There were qualitative differences between patients in terms of how they responded to being given lifestyle advice, their readiness to make change and the emotions they expressed about their illness and behaviours.

Some patients arrived expecting lifestyle advice and expressed feeling in control of this process. For these patients, the main support needs related to keeping up motivation—they wanted reminders, help with monitoring, emailing or providing follow-up information and prompts. Support was often related to easing the cognitive burden of remembering, as well as providing social, person-to-person encouragement. For these patients, lifestyle recommendations were seen as part of their treatment, seemed to promote a sense of control and was often acted upon.

Others, particularly those who expressed more stress and negative emotional associations with illness (or with specific behaviours), did not always respond well to being given advice, seeming unready for making changes. They were more overwhelmed by symptoms, more stressed or fearful about illness, and felt a lack of control regarding their health. One practitioner explained in our interview how she considered supporting lifestyle change in the context of the patients' current stress levels:

*Practitioner Susan: “If they are going through a super stressful period and they are smoking 20 a day then I wouldn’t always necessarily think that right then and there is the time for them to stop smoking I kinda think well let’s get through the stress and let’s look at other things and when the stress has gone a bit less they are much more likely to quit smoking”.*

These patients were not so much unmotivated to make changes, but at a different stage where motivation was not possible or relevant until emotional regulation, stress reduction and/or symptom improvement had occurred. Participants described these changes as increasing their sense of control over their health and, for some, greater readiness to consider lifestyle changes.

For example, patient Sam had felt very distressed about her illness, unable to get a diagnosis or to engage with her usual levels of physical activity. During her interview, Sam described improvements in her outlook which appeared to increase her sense of capability and readiness to re-engage with activities she had previously avoided.

*Patient Sam: “I kind of started to feel more positive. Not down all the time and then I started to kind of remind, just started to reach out to how I used to be. Now that used to get me really down ‘cause, you know when you're an active person and...but then I kind of thought well you know I can do a little bit, try and do a little bit, so started doing a little bit of walking and then now I'm doing a little bit more.”*

For patient Gillian, the first suggestion of lifestyle change (to increase physical activity) was too early and she explained why she felt unable to think about this change because of her symptoms and stress.

*Patient Gillian: “She asked, ‘Do I exercise and get a walk?’ Or ‘how often I could do about 10 minutes?’ She said, ‘Could you extend it to about 20 minutes?’ I said ‘No’, I have to do what I want to do. If I push myself and it becomes a chore, I need to be able to, at the moment…I have really gone through an emotional roller coaster. And right at the moment, what I want to do is what I want to do... I actually struggle putting one foot in front of... Probably about three weeks ago, I thought it was very, very tough. So I just do what I want to do. That makes me happy. And I find moments of reflection and happiness where I can”.*

By our second interview, Gillian described improved emotional regulation and reduced distress. At this stage she appeared more receptive to discussions about lifestyle change, although this did not necessarily translate into immediate behaviour change. However, not all patients moved through this process. One patient with serious chronic health issues did not feel much benefit from treatment, experienced little improvement in symptoms and felt unable to make changes to lifestyle. Their current symptom concerns left them with little capability or motivation to engage with behaviour change. This highlights how difficult engagement with behaviour change support can be for some patients when distressing symptoms remain unresolved.

Many of the patients expressed the importance of autonomy, preferring language and approaches from practitioners which provided non-judgemental, optional suggestions.

This theme indicates the importance of initiating lifestyle discussions in a manner and time which corresponds with and supports patients' current level of capability and motivation. Whereas several patients seemed to gain control through their practitioners' advice, others felt unable to discuss lifestyle change, viewing any lifestyle change as burdensome.

### Trust and acceptance: in practitioner, in explanations, in treatment

Patients frequently described their willingness to consider lifestyle changes as linked to their acceptance of treatment. There was a particular emphasis on the importance of trusting the therapist and having a good relationship, but there was also trust in the acu-point needling treatment (particularly if they felt this treatment improved symptoms or elicited other benefits like stress reduction) and trust in the practitioner's explanations of illness, treatments and connections to lifestyle factors.

#### Trust in therapist

Therapeutic relationships developed over long consultations, with time to discuss illness, concerns and feelings in depth. There was often broad discussion of the patient's personality and patterns of behaviour. These lengthy and deep discussions fostered trust in the traditional acupuncturist.

*Patient Stephanie: “I think because of the length of time we're able to discuss basically my lifestyle and what I wanted to achieve and things like that, that made a massive difference. For example, if I was to go to a doctor, it would be a 10 minute appointment. It would be rushed. I wouldn't get much detail, they probably wouldn’t actually provide much information because it was just the actual quality of the time that we spent discussing the options for me and the follow up as well.”*

#### Trust in explanations

Some patients' understanding of the relationships between their symptoms/physical states and their lifestyle/behaviours developed through the explanations offered by traditional acupuncturists. The patients described how during their discussions they were able to understand these links, either between symptoms and behaviours (e.g. sore throat and smoking), or between environmental/social factors and health behaviour (stressful job/long hours preventing engaging in healthy activities). The explanations offered were frequently (though not always) informed by their specific East Asian/Chinese medical framework, where illness categories are seen as broad patterns (often including both physical and psychological aspects).

*Cara Consultation:*

*Practitioner: “Yeah, I think like, when we're looking at anything that's to do with rhythm, and fertility is one of those things along with...blood sugar, and sleep. It's just very helpful to kind of address all the other rhythmic things in our lives”.*

Some patients found these explanations meaningful because they offered new ways of understanding their health and links to behaviours; this was most striking when symptoms had been medically undiagnosed. Patients sometimes expressed a strong need to make sense of illness experiences, feeling discomfort with ambiguity/not knowing. When patients found these explanations meaningful, they often appeared more receptive to advice and discussion about lifestyle change.

*Isobel Consultation:*

*Practitioner: “One of ‘Wood’s’^*1*^ jobs in the body is to smooth all the body processes, so when you were talking earlier on about drinking [water] and exercising and then having better digestion, that's because ‘Wood’ likes to move and if everything, if ‘Wood’ is happy, all of your body processes will be smooth and that's everything from periods going smoothly, digestion going smoothly, bowels, breathing, the whole lot, it does the whole lot.”*

In her interview, patient Isobel explained how much she had connected with these explanatory frameworks.

*Patient Isobel: “ it just makes so much sense and I love science anyway and I understand there's a bit, it's a fine line between, you know, ancient Chinese medicine and actual scientific proof. But a lot of it makes complete sense, just taught me more than just acupuncture.”*

Practitioners also offered explanations to patients which connected symptoms, broader health patterns and behaviours in a straightforward way. For example, Amelie and her practitioner connected smoking with her current congestion issues:

*Practitioner “I suppose it seems quite relevant to this congestion.”*

*Patient Amelie “I definitely noticed like it's worse when I smoke”*

*Practitioner: “...with this congestion, throat, sinuses, kind of a feeling that things have got stuck and lodged in your head. Smoking would be the first thing that I would say it's like, let's clear that out of the way.”*

*Patient Amelie: “Yeah, definitely”.*

#### Trust in treatment

When patients felt better after treatments, they often described being more open to lifestyle suggestions and more motivated to engage with recommended changes. The patients who felt they were getting benefit from the acu-point needling appeared to feel a stronger connection to the therapist and have more trust in the whole intervention (including in the lifestyle/behaviour change aspects).

In her second interview Isobel talked about how the treatment had helped her to make some changes in her life, change her job, find more time to exercise and begin to socialise again. She described how the explanations, the experience of feeling benefit from the treatment and from the lifestyle change had reinforced the lifestyle changes:

*Isobel “Uh, I mean, I probably did a few of the bits anyway, but you know, when you’re told it works. And then you see it works directly. You're more likely to go back to it rather than a fluke of doing it and then you feel better, you know, I mean?”*

In another interview with patient Amelie, she explained what it was about the sessions she had found helpful in supporting her to make behaviour changes and described how her experience of the treatment working was important.

*Patient Amelie “Yeah, no, there's definitely like a few, especially like right after I leave the treatment. It just feels like so refreshed and good. I think I struggled a lot with stress because of school because it's obviously this is my 4th year. So I had, I was like trying to finish everything and having the sessions like I really I think relieved a lot of my anxiety. I think it would have been a lot worse....Yeah, definitely. I think I think it really like calms me down and it allows me like I don't know how to say this like it gives me kind of like the space to work on those things if that makes sense.”*

Participants' accounts suggested that trust in the practitioner, explanations and treatment experiences often developed together and may have contributed to acceptance of lifestyle advice.

### Changes in feelings: mood, calm, positivity and ‘just feeling like it’

Several patients described a connection between feeling well during treatment and increased motivation or readiness to engage with lifestyle changes. Patients described tuning into their physical states during treatment, feeling ‘relaxed’, ‘positive’ and ‘lifted’ during or immediately after treatment. This was often attributed to acu-point needling and sometimes to the therapeutic relationship (e.g. feeling listened to). These patients described treatment experiences as generating feelings of wellness which appeared to increase their openness to healthier behaviours.

In her third treatment, Gillian told the practitioner how she felt immediately after her previous treatment.

*Patient Gillian: “I really felt after you treated me last time a great calmness…a really good calmness. I really did and it was notable.”*

A few weeks later in our interview, Gillian explained how the feeling of her mood being lifted during the treatments had been important to her and appeared to increase her sense of capability and willingness to re-engage with activities she valued.

*Patient Gillian: “And when these lifting of my mood, she put the needle to the top of my head on four occasions. And that really felt lifted. So much so yeah, that's good thing, isn't it? …couldn't come at a better time. Yeah, it couldn't have been more productive in myself and wanting to be more proactive. I've been literally living almost half days. Yeah. Now, you know I. I'm. I'm really getting on and with it”.*

In Amelie's interview, she also described how important the experience of acu-point needling treatment had been in helping her to connect to a feeling of wellness, and how this contributed to her motivation to maintain healthier lifestyle choices.

*Patient Amelie: “I don't know more of just like how good you feel after it and then not wanting to mess that up because I don't know every time you smoke you kind of just feel like a little bit more like gross in your body. Yeah, you know...I think like, especially when you're in the acupuncture treatment and you sit there and you kind of go into this like meditative state. I like it. I'm just like ‘Oh yeah, I forget how good this feels and I definitely wanna keep that up’”.*

Patients frequently expressed being guided by feelings, both as a motivating factor to change but also as a reason for not engaging in healthy behaviours (‘just not feeling like it’).

*Patient Gillian “I do have a treadmill and I try and do at least 10 minutes a day”*

*Practitioner “It’s pretty good 10 minutes a day. Would you consider taking that to 15?”*

*Patient Gillian “Not at the moment...If I was taking 15 minutes a day, I wouldn't use it. And I have to use it. So let's just do what I feel happy with. Because life it’s got to be worth living. I mean, I'm in that mode at the moment”*

Both situations suggest that participants often described feelings as relevant to decisions about health behaviours. Treatment experiences were sometimes associated with changes in mood, calmness or awareness, which participants described as increasing their readiness to engage with healthier behaviours. However, greater readiness did not always correspond with immediate behaviour change.

### Negative and partial change cases

Several participants described increased understanding of lifestyle factors, improved mood, or reduced stress without substantial behaviour change. For some, symptom burden remained the dominant concern and behaviour change was not prioritised. Others expressed ambivalence about lifestyle recommendations, accepting some suggestions while declining others. These cases suggest that greater readiness or openness to change did not necessarily lead to immediate enactment of behaviour change.

## Discussion

This in-depth longitudinal study provided insight into the way support for behaviour change was provided and experienced by patients and suggests some key themes regarding the process of health behaviour change in the novel setting of traditional acupuncture practice. Each of the three main themes will be discussed below in relation to relevant behaviour change theories that may offer useful interpretive frameworks for understanding the findings. The findings suggest that patients experience support for behaviour change in direct and indirect ways: directly through receiving individualised advice about behaviour, and indirectly through feeling supported in a therapeutic partnership or having symptoms treated which may be barriers to health behaviours. This links with previous literature which has highlighted the role of the therapeutic relationship in supporting change (Lie et al., 2017; Polley & Sabey, 2022) and the importance of individualised advice (Evans et al., 2011). This support may be experienced explicitly e.g. through gaining better understanding of the relevance of health behaviour to one's own health, and implicitly e.g. through feeling in a more positive/more receptive mood.

### Implications for behaviour change theory

As shown in our theme ‘From Gaining Control to Keeping up Motivation’, different kinds of support were suitable for patients at different stages of the change process; in particular, patients with high levels of stress/emotional dysregulation or overwhelming symptoms responded better to support for stress and symptom reduction either before or alongside behaviour change support. This finding can be understood in relation to the Trans-Theoretical Model and Stages of Change (TTMSoC) (Prochaska & DiClemente, 1983). Different stages in the change cycle may be associated with different experiences, needs and barriers to change. In this study, some participants appeared to resemble individuals described as being in pre-contemplation and seemed to place more emphasis on capability, reducing stress and prioritising improvement of challenging symptoms. Some patients who appeared to be in a contemplation-preparation stage placed more value on support which emphasised motivation and practical help (reminders, monitoring, specific information and advice on relevant lifestyle changes).

We found that some patients felt better able to consider and make lifestyle change after their stress/symptoms had improved, suggesting support at this stage of the change cycle could benefit from a wider focus to address stress and capability. There may also be other barriers to change in this stage, such as lack of therapeutic alliance or any number of social/environmental factors.

The finding in this study that patients' level of stress concerning their health corresponded with a lack of control to make behaviour changes can be interpreted through aspects of Social Cognitive Theory (SCT) (Bandura, 1991). SCT underlines the role of self-regulation in behaviour change and recent research has suggested both the important role of self-regulation as a mediator of the effects of health behaviour change interventions, and the role of mood as a moderator of intervention effect on self-regulation (Stoeckel et al., 2017). Improvement in anxiety, depression and negative mood states have been found to moderate self-regulation increase in weight-loss treatments (Annesi, 2017), perhaps through increasing the emotional and cognitive resources available for behaviour change. Although the present qualitative study did not assess these constructs directly, the participants' accounts appear broadly consistent with this literature.

As shown in our theme ‘Trust and Acceptance: In Practitioner, In Explanations, In Treatment’, our analysis suggests that trust in different elements of traditional acupuncture (practitioner, explanations and treatment experiences) developed together and may help explain participants' acceptance of behaviour change support.

The central importance of trust in the practitioner fits with previous research on the role of trust within the therapeutic relationship (Birkhäuer et al., 2017; Greene, 2022; Murray & McCrone, 2015; Ward, 2018). Participants often described perceived treatment benefit as strengthening their trust in the practitioner, explanations and advice offered. Similar findings have found patients' trust in their TCIM therapist predicts adherence to follow-up appointments (Bishop et al., 2008). Furthermore, patients' perception of whether lifestyle advice is a part of treatment has been found to increase acceptance (Bergen, 2020).

One possible interpretation is that explanations perceived as meaningful may have contributed to greater illness coherence, a common sense model of self-regulation (CSM) construct referring to a patient's clarity in understanding their illness (Moss-Morris et al., 2002). Although illness coherence was not directly assessed in this study, participants frequently described valuing explanations that helped them make sense of their symptoms. This meta-cognitive belief about illness is thought likely to have pervasive effects on patients' coping responses to illness (Hagger & Orbell, 2022). Positive beliefs about control over illness and stronger illness coherence are associated with more adaptive behavioural coping responses (e.g. adherence to treatment) (Hagger et al., 2017). Importantly, our findings do not suggest that participants adopted these explanations because they were empirically validated biomedical accounts. Rather, participants frequently described them as meaningful frameworks that helped them make sense of their experiences and the relationship between symptoms and lifestyle.

As shown in our theme ‘Changes in Feelings: mood, calm, positivity and just feeling like it’, the guiding role of patients' feelings in making decisions about behaviours was an important theme. Patients' explanations of their changed behaviours were sometimes based on their feelings and shifts in mood. While stress reduction and better mood may be a moderator of self-regulation skills (discussed above) changes in feelings may be relevant to understanding participants' decisions about behaviour change.

Dual process theories of behaviour have suggested two decision-making processes which work together (one non-rational and one rational). The non-rational system is thought to be based on experience and associations as opposed to reflective thoughts (Hagger, 2016; Sheeran et al., 2013) and evidence is growing of the role of these processes in predicting behaviours (Rebar et al., 2016). Non-rational decision-making processes may also be moderated by physical experiences/changes: for example, increased interoception (awareness of physiological processes) moderates emotional/intuitive decision-making, and bodily responses influence aspects of emotion experience and decision-making (Dunn et al., 2010). These processes may offer one interpretation of our finding that some patients expressed their reasons for making changes in lifestyle as being connected to feeling well, calm, and/or relaxed during their treatment sessions.

We also found that patients expressed that they did or didn't make certain changes because they ‘felt like it’ or ‘didn't feel it’. These findings concerning decisions based on feelings suggest one possible perspective on why intentions and behaviour do not always align. Research into epistemic or noetic feelings (‘feelings of knowing’) (Arango-Muñoz, 2014; Koriat, 2000) suggests that sensations about mental states may be more directly responsible for decisions than deliberative reasoned thought. Participants' accounts suggest the possible relevance of emotions, mood and heuristics in decisions about health behaviours.

## Strengths and limitations

Using multiple sources of data (consultations and interviews with patients and practitioners) over a period of several weeks starting from the initial consultation allowed analysis to explore processes of change longitudinally over time, providing a deep level of insight into how changes occurred.

This study is limited by the small homogenous sample (involved three traditional acupuncturists and nine patients, only 1 male, all based in the UK), self-selecting participants, private-paying sample, and the lack of other sociodemographic information including ethnicity and education level. The acupuncturists in this study were selected partly on the basis that they had previously reported engaging in behaviour change support. In self-selecting this treatment, these patients may be more open than typical patients to the treatments and explanatory frameworks of traditional acupuncturists; however, this reflects usual practice and there was a fair amount of variability in patients' interest/prior knowledge of traditional acupuncture. Further, it was not clear which patients would embrace treatment/explanations from the outset, and it was not unusual for patients to change their perspective during the treatment. Since the treatments were provided privately, outside the National Health Service (NHS), all patients were both capable of paying for treatments and had made the commitment to paying for treatment (i.e. were somewhat invested). Findings may not be transferable to publicly funded acupuncture settings or acupuncture clinics where practitioners rarely engage in health behaviour change support.

There was also an explicit exclusion of non-English language patients and substance and alcohol dependence disorders, which limits transferability to these groups. There is a need for future work to consider underserved/disadvantaged patient populations. It is also possible that findings were limited by social desirability in behaviour-change accounts; we found most of the patients were relatively motivated to make behaviour changes from the outset, which is not necessarily typical for other patients being offered behaviour change support. However, this was not the case for all patients: some were not expecting or motivated to make behaviour changes, and some participants did talk about finding behaviour change difficult and not doing it. Our findings are also limited by the relatively short duration of the study, and we do not have long-term follow-up data to understand how or whether behaviour changes were maintained after treatment finished. The study is limited due to a lack of patient involvement in the design or management of the study.

## Conclusion and implications

Patients receiving support for lifestyle/behaviour change within traditional acupuncture treatments valued different types of support according to their needs: Acu-point needling/stimulation, which patients felt helped with obstacles to change such as pain symptoms and stress; Therapeutic trust, and providing alternative biopsychosocial explanations were valuable, as were the positive feelings often experienced during/after treatments. These potential facilitators of change should be explored further to establish mechanisms of action and could be used to update the logic model of behaviour change in traditional acupuncture previously proposed (Pinto et al., 2021). This work could generate testable hypotheses concerning relationships between the components of this intervention, mediators, moderators and outcomes. Further exploratory work could also be undertaken to explore the role of these components in similar individual clinical interventions for supporting behaviour change. Larger scale trials or other intervention evaluations could establish the effectiveness of the approach of traditional acupuncturists in supporting behaviour change. While this work is at an exploratory stage, ultimately if these approaches are found to be effective, they may be integrated into health promotion strategies alongside current mainstream approaches (such as brief intervention techniques and social prescribing used in the NHS).

There are further possibilities for cross-disciplinary research collaboration, with traditional acupuncturists potentially benefitting from training in psychological techniques (such as behaviour change techniques (BCTs) and motivational interviewing), and further benefits from psychological research exploring traditional acupuncture, which may provide new insights into behaviour change methods and theory.

## Supplementary Material

SupplementaryFile4PatientConsentformNotReview.doc SupplementaryFile4PatientConsentformNotReview.doc

SupplementaryFile3AcupuncturistConsentformNotReview.doc SupplementaryFile3AcupuncturistConsentformNotReview.doc

SupplementaryFile2ParticipantInformationPatientsNotReview.docx SupplementaryFile2ParticipantInformationPatientsNotReview.docx

SupplementaryFile6InterviewTopicGuidPatientsNotReview.docx SupplementaryFile6InterviewTopicGuidPatientsNotReview.docx

SupplementaryFile5InterviewTopicGuideAcusNotReview.docx SupplementaryFile5InterviewTopicGuideAcusNotReview.docx

SupplementaryFile1ParticipantInformationAcusNotReview.docx SupplementaryFile1ParticipantInformationAcusNotReview.docx

## Figures and Tables

**Table:** 

(a) Institutional Review Board Statement: The study was conducted in accordance with the Declaration of Helsinki and was approved by an Institutional Review Board/Ethics committee. See details under Methods.	X
(b) The study received an exemption from an Institutional Review Board/Ethics committee; See details under Methods.	

## Data Availability

The data are not publicly available due to privacy or ethical restrictions (we did not receive consent to share the data).
