# Supplementary material for: Exploring the process of making health behaviour changes in traditional acupuncture: a longitudinal qualitative study
Source: Health Psychol Behav Med. 2026 Jul 28;14(1):2709724. doi: 10.1080/21642850.2026.2709724 (PMC13417641; doi:10.1080/21642850.2026.2709724)
Supplement: SupplementaryFile3AcupuncturistConsentformNotReview.doc [file RHPB_A_2709724_SM0016.doc]

**ACUPUNCTURIST CONSENT FORM**

**Study title**: Lifestyle and health behaviour change outcomes in traditional acupuncture. A qualitative study

**Researcher name**: xxx

***Please initial the box(es) if you agree with the statement(s):***

| I have read and understood the Acupuncturist Participant Information form (29/10/2021: Version 1) and have had the opportunity to ask questions about the study. |  |
| --- | --- |
| I agree to take part in this research project and agree for my data to be used for the purpose of this study. |  |
| I understand that my patients’ acupuncture treatment consultations will be audio-recorded and the data will be used for the purposes of this study. |  |
| I understand that taking part includes participating in one semi-structured interview with the researcher which will be audio recorded for the purposes of this study. |  |
| I understand my participation is voluntary and I may withdraw for any reason without my participation rights being affected. |  |
| I understand that should I withdraw from the study then the information collected about me up to this point may still be used for the purposes of achieving the objectives of the study only. |  |
| I understand that I may be quoted directly in publications of the research but that I will not be directly identified (e.g. that my name will not be used). |  |

Name of participant (print name)……………………………………………………………………………

Signature of participant……………………………………………………………………………………….

Date……………………………………………………………………………………….. ………………….

Name of researcher (print name)……………………………………………………………………………

Signature of researcher ……………………………………………………………………………………….

Date………………………………………………………………………………………………………………..
