# Supplementary material for: Exploring the process of making health behaviour changes in traditional acupuncture: a longitudinal qualitative study
Source: Health Psychol Behav Med. 2026 Jul 28;14(1):2709724. doi: 10.1080/21642850.2026.2709724 (PMC13417641; doi:10.1080/21642850.2026.2709724)
Supplement: SupplementaryFile2ParticipantInformationPatientsNotReview.docx [file RHPB_A_2709724_SM0017.docx]

**Participant Information Sheet (Patients)**

**Study Title**: **Lifestyle and Health Behaviour Change outcomes in traditional acupuncture. A qualitative study**

**Researcher**: xxx

You are being invited to take part in the above research study. To help you decide whether you would like to take part or not, it is important that you understand why the research is being done and what it will involve. Please read the information below carefully and ask questions if anything is not clear or you would like more information before you decide to take part in this research. You may like to discuss it with others but it is up to you to decide whether or not to take part. If you are happy to participate you will be asked to sign a consent form.

**What is the research about?**

This qualitative study is being undertaken to develop an understanding of good practice for acupuncturists in regard to supporting patients with making lifestyle and health behaviour changes, such as increasing exercise, improving eating habits, reducing smoking or alcohol consumption.

The study will explore the experiences of both acupuncturists and patients in relation to lifestyle and health behaviour change. Your acupuncturist has been asked to recruit 3 patients to take part in the study. The research will involve making audio recordings of your treatment consultations and conducting one research interview with your acupuncturist and two research interviews with you.

**Why have I been asked to participate?**

You are being invited to take part because your acupuncturist has agreed to be in this study.

**What will happen to me if I take part?**

If you take part, I would like to interview you on two occasions. The first interview will be after you have had two acupuncture appointments, the second interview will be approximately 6 weeks later. Each interview will last approximately 60 minutes and will take place online or in person (your choice). During the interviews, I will ask you some questions about your experiences of acupuncture and particularly about any areas of lifestyle change that were discussed during your treatments. The interviews will be ‘qualitative’, which means there are no right or wrong answers, and I am interested in hearing all about your experiences, thoughts, and feelings in your own words. I will also ask your acupuncturist to audio record your acupuncture treatment consultations so that I can listen to them. I will also interview your acupuncturist about their approach to acupuncture consultations.

The recordings of the consultations and interviews will be analysed together and used to develop an understanding of good practice for supporting lifestyle and health behaviour change in traditional acupuncture patients.

If you agree to take part you will be asked to sign and return the Participant Information and Consent Form by email.

**Are there any benefits in my taking part?**

Each participant in this study (both acupuncturists and patients) will be offered a £20 voucher on completion of the final interviews. There is also the wider benefit of being involved with this research to develop professional standards and improve patient outcomes.

**Are there any risks involved?**

The subject matter of this study will include your personal health habits, such as diet, exercise, alcohol consumption, smoking, stress management or work/social life. If you feel uncomfortable about answering any of these questions you are entitled to withdraw consent or skip questions whenever you wish (I will remind all participants again at the beginning of interviews that consent can be withdrawn or questions skipped).

**What data will be collected?**

I will be collecting data in the form of audio-recordings, written notes and your completed consent form. I will send digital audio equipment directly to your acupuncturist, in order for them to make audio recordings of the treatment consultations with participating patients. I will provide a secure server address for acupuncturists to upload audio files via Safe-send (or arrange a courier to collect the equipment and recordings). All digital files including recordings of consultations and interviews will be stored on secure research drives at Xxx University. Hand-written notes will be destroyed after putting data onto files which will be saved onto secure research drives at Xxx University (and no personal or contact information will be stored with or linked to this data). If you supply any additional contact details during the study these will be kept separately from the research data, saved on secure research drives at Xxx University, and deleted after you have taken part in the study and have been paid incentives.

**Will my participation be confidential?**

Your participation and the information collected about you during the course of the research will be kept strictly confidential. Only members of the research team and responsible members of the University of Xxx may be given access to data about you for monitoring purposes and/or to carry out an audit of the study to ensure that the research is complying with applicable regulations. Individuals from regulatory authorities (people who check that we are carrying out the study correctly) may require access to your data. All of these people have a duty to keep your information, as a research participant, strictly confidential.

**Do I have to take part?**

No, it is entirely up to you to decide whether or not to take part. If you decide you want to take part, you will need to sign a consent form to show you have agreed to take part**. If you agree to take part please reply to me, xxx**

**What happens if I change my mind?**

You have the right to change your mind and withdraw at any time without giving a reason. If you withdraw from the study after two weeks from completion of consultation recordings or interviews (during the data analysis stage) we will keep the information about you that we have already obtained for the purposes of achieving the objectives of the study only as this information will be impossible to disentangle from the other data. Please contact xxx if you decide to withdraw.

**What will happen to the results of the research?**

Your personal details will remain strictly confidential. Research findings made available in any reports or publications will not include information that can directly identify you without your specific consent.

The results will be used to develop an understanding of good practice for achieving lifestyle and health behaviour changes. The results may be included in my PhD thesis and future publications.

**Where can I get more information?**

The research team can be contacted if you have any further questions:

Xxx

**What happens if there is a problem?**

If you have a concern about any aspect of this study, you should speak to the researchers who will do their best to answer your questions:

xx

If you remain unhappy or have a complaint about any aspect of this study, please contact the University of Xxx Research Integrity and Governance Manager xxx

**Data Protection Privacy Notice**

The University of Xxx conducts research to the highest standards of research integrity. As a publicly-funded organisation, the University has to ensure that it is in the public interest when we use personally-identifiable information about people who have agreed to take part in research. This means that when you agree to take part in a research study, we will use information about you in the ways needed, and for the purposes specified, to conduct and complete the research project. Under data protection law, ‘Personal data’ means any information that relates to and is capable of identifying a living individual. The University’s data protection policy governing the use of personal data by the University can be found on its website xxx

This Participant Information Sheet tells you what data will be collected for this project and whether this includes any personal data. Please ask the research team if you have any questions or are unclear what data is being collected about you.

Our privacy notice for research participants provides more information on how the University of Xxx collects and uses your personal data when you take part in one of our research projects and can be found at xxx

Any personal data we collect in this study will be used only for the purposes of carrying out our research and will be handled according to the University’s policies in line with data protection law. If any personal data is used from which you can be identified directly, it will not be disclosed to anyone else without your consent unless the University of Xxx is required by law to disclose it.

Data protection law requires us to have a valid legal reason (‘lawful basis’) to process and use your personal data. The lawful basis for processing personal information in this research study is for the performance of a task carried out in the public interest. Personal data collected for research will not be used for any other purpose.

For the purposes of data protection law, the University of Xxx is the ‘Data Controller’ for this study, which means that we are responsible for looking after your information and using it properly. The University of Xxx will keep identifiable information about you for 10 years after the study has finished after which time any link between you and your information will be removed.

Your acupuncturist will keep identifiable information about you from this study until the data collection has finished then will delete any information about you in relation to this study.

To safeguard your rights, we will use the minimum personal data necessary to achieve our research study objectives. Your data protection rights – such as to access, change, or transfer such information - may be limited, however, in order for the research output to be reliable and accurate. The University will not do anything with your personal data that you would not reasonably expect.

If you have any questions about how your personal data is used, or wish to exercise any of your rights, please consult the University’s data protection webpage (https://www.xxx.ac.uk/legalservices/what-we-do/data-protection-and-foi.page) where you can make a request using our online form. If you need further assistance, please contact the University’s Data Protection Officer xxx.

**Thank you** for taking the time to read this information sheet and considering taking part in the research.
