# Supplementary material for: Exploring the process of making health behaviour changes in traditional acupuncture: a longitudinal qualitative study
Source: Health Psychol Behav Med. 2026 Jul 28;14(1):2709724. doi: 10.1080/21642850.2026.2709724 (PMC13417641; doi:10.1080/21642850.2026.2709724)
Supplement: SupplementaryFile6InterviewTopicGuidPatientsNotReview.docx [file RHPB_A_2709724_SM0018.docx]

**Interview Topic Guide (Patients)**

A topic guide has been devised consisting of 11 primary questions, around the participant’s experience of lifestyle and health behaviour change with both covert and overt questions that aim to capture revealing data:

1. What were your reasons for seeking acupuncture treatment?
2. What were you hoping to get from treatment?

I think that you talked with your acupuncturist about XXX lifestyle/health behaviours, how did you feel about that? *(prompts refer to changes identified in consultation recording e.g: dietary changes, increased physical activity, sleep hygiene practices, smoking reduction, alcohol reduction , mindfulness practices/stress reduction techniques (e.g. meditation, tai chi, qigong), rest/relaxation (e.g. hobbies, spending time outdoors), change to work/social habits.)*

1. What changes if any were you able to make?
2. How were the lifestyle changes agreed/decided?
3. Was there anything about the acupuncture sessions you found helpful for supporting these changes?
4. Was there anything about the acupuncture sessions that you found unhelpful in supporting your lifestyle changes?
5. Was there anything about the acupuncture sessions that could have been done differently to help support your lifestyle changes?
6. Were you able to continue with the changes over time?
7. Was there anything that made it difficult for you to make the changes you’d talked about?
8. What else you can tell me about this subject?
