# Supplementary material for: Exploring the process of making health behaviour changes in traditional acupuncture: a longitudinal qualitative study
Source: Health Psychol Behav Med. 2026 Jul 28;14(1):2709724. doi: 10.1080/21642850.2026.2709724 (PMC13417641; doi:10.1080/21642850.2026.2709724)
Supplement: SupplementaryFile5InterviewTopicGuideAcusNotReview.docx [file RHPB_A_2709724_SM0019.docx]

Interview Topic Guide (Acupuncturists)

A topic guide has been devised consisting of 10 primary questions, around the participant’s experience of supporting lifestyle/health behaviour change:

1. How did you feel about supporting lifestyle or health behaviour changes for [patient xxx]?
2. What role do you think lifestyle/behaviour change plays in the treatment? *(if needed prompt for patients 1/2/3)*
3. How did you choose which lifestyle/behaviours to work on changing? *(prompt patient 1/2/3)*?
4. How did the topic of lifestyle/behaviours come up?
5. What guided your decision about when or whether to discuss lifestyle/behaviours?
6. How were specific lifestyle changes agreed/decided?
7. How did you explain lifestyle/behaviour change? (Prompt – the need/the role for lifestyle/behaviour changes)
8. How do you think (patient 1/2/3) responded to making these changes? (*Refer to changes identified in consultation recording/previous questions for prompts*)
   1. Was there anything you think (patient 1/2/3) found particularly helpful in regard to making changes?
   2. Was there anything that you think prevented (patient 1/2/3) from making changes?
   3. What do you think is the key to patients (prompt patient 1/2/3) keeping up the changes?
9. How typical were the consultations with patient xxx of your usual practice?

9a) Possible prompts - what kinds of lifestyle changes are part of your usual advice and which are specific to this patient? Was there anything unusual about patient xxx?

1. What else you can tell me about this subject?
